# Introductory Lecture. Ohio College of Dental Surgery

**Published:** 1877-11

**Authors:** J. S. Cassidy

**Affiliations:** Prof. of Chemistry


### INTRODUCTORY LECTURE.

*To the Class in the Ohio College of Dental Surgery, Oct.* 18, 1877.

BY J. S. CASSIDY, M. D., D. D. S., PROF. OF CHEMISTRY.

*Gentlemen*—It is not only proper but expected, that on this occasion, the first of a long series of similar meetings, we should lay aside the formality of presenting a regular lesson with its unavoidable concomitant suspense of expected quiz, and occupy our time, perhaps more pleasantly, with a brief review of some sadly existing ambiguities of chemical nomenclature.

"And the Lord God having formed out of the ground all the beasts of the earth, and all the fowls of the air, brought them to Adam to see what he would call them; for whatsoever Adam called any living creature, the same is its name."

Names are given to all known objects as a necessary convenience, to distinguish the individuality of each, and the important first step in acquiring a new acquaintance, is to learn the designation of the subject, thing, or person introduced.

In the nomenclature of those subjects called scientific, we generally find the terms simple and extremely well suited to the purpose for which they were applied; in most cases each department is so systematized that duality of terms for any given object is avoided. Our own department, alas, proves the rule by the exceptional confusion of its language when referring to the multiplicity of appellations, which many of its best known compounds have received.

When chemistry by the discovery of oxygen, emerged from the gloaming that surrounded the unrequited labors of the alchemists, each new elementary form of matter, as ascertained to be practically simple in its nature, was, in most instances happily, designated according to possession of real or fancied suggestive physical property, or in honor of somebody or something previously known. Thus "oxygen" from *oxus* and *Genesis*, Greek words signifying "acidmaker," because it was supposed to be a necessary constituent of all acids; and "hydrogen" similarly derived, from *huor*-water and *genesis*, because of its presence to the formation of water (H. and O.) "Selenium" the *moon*, not that this substance resembled in the least, the silvery queen of night, but as the *earth-tellus*, already being honored by "tellirium," its satellite must also be commemorated, by the next of kin to the latter element. "Chlorine," *pale yellow*," "iodine" *violet like*, "bromine" by its *bad odor* and "fluorine" *to flow* from one of its compound *fluor spar*; "Sodium" from *sod ash*, obtained by burning sods of marine plants; "potassium," the metallic radicle of *potash* the latter derived by evaporating lixiviated wood ashes in *pots*, "kalium" the other synonym of this element from *kali* Arabic for ashes, and hence also the derivation of the word "alkali." A certain compound was first made by the Arabs of the desert of Libya, from camels dung, which they called "sal ammoniac" or "ammon salt," in honor of the neighboring temple of Jupiter *Ammon*, hence the hypothetical metal "Ammonium" (N H 4.) The name "antimony" is curiously said to be derived from *anti* against, and *moine*, French for *monk* from the fact that Basil Valentine a celebrated German monk sickened all of his immediate brethren in an earnest desire to ascertain the medicinal properties of this element. Of the few metallic elements with which the ancients were acquainted, we may mention "mercury" so named after the messenger of the gods on account of its rapidity of movement, and "hydrargyrum" from Greek *huor-water* and arguros, "silver" from *argos* "white" suggested the silvery synonym "argentum." "Gold" an old saxon word signifying *yellow*, and "aurum" its Latin name from a Hebrew word expressive of the *color of fire*. "Copper" first worked on the island of Cyprus in the Mediterranean, hence "cyprium" "cuprum" "copper."

And so we might proceed giving reasons for the etymology of all the elements, down to the last claimed discovery of a new metal by Sergius Kern of St. Petersburg, while separating rhodium and iridium from platinum ores. With characteristic generosity he has named the chemical infant "Davyum, "in honor of the great English chemist Sir Humphrey Davy, and let me say parenthetically, that no greater posthumous honor could be conferred than this; neither "storied urn nor animated bust" imparts a tithe of the proud distinction, which by this graceful act, encircles the memory of the worthy dead.

A few years subsequent to the discovery of oxygen an attempt was made to systematize the nomenclature of the elements so as to signify for each, its leading chemical property; but confusion was found to be the consequence; some of them being familiar substances as sulphur, gold, iron and other metals well known in the arts, were almost of necessity allowed to retain the names they bear in common language. This difficulty, if such it be, was partially overcome by affixing a similar termination to those elements that resembled each other in some particular property as *chlorine*, brom*ine*, iod*ine* and fluor*ine*, oxy*gen* hydro*gen* and nitro*gen*; and so also more notably the effort to affix the terminal syllable *um* to the names of the metals, by substituting the Latin synonym for the one ordinarily used; aur*um* for gold, fer*um* for iron etc., and to those not found in nature in the metallic state, the letter *i* was inserted as indicative of the fact, as in kalium, aluminium, etc.

It would appear an easy matter to follow such a simple rule step by step, and give to each new *substance*, as introduced into the family of elements, a suggestive name with its proper termination; but alas for consistency, it was not uniformly possible to determine the sex of the youthful stranger, so the few supposed hermaphrodites were placed on the metallic side of the house, in which position—although now known to be non-metallic, they remain by virtue of still retaining their original appellation. Thus *silicium* (occasionally silicon) *selenium* and *tellurium*, the latter, two especially forming, *acid* instead of *basic* oxides, corresponding to those of non-metallic sulphur. Of the true metals, there are five whose names are devoid of the conventional termination, nickle, zinc, cobalt, tungsten, or *wolfram*, rarely *wolframium*, and bismuth, the first of these having completely erased the stigma of falsehood implied by its German christening "kupfer-nikel." We could also criticise the orthography of *davyum*, wherein the vowel *i* might be substituted for *y* and so preserve the idea of system without changing in the least the evident origin of the name.

The limited number of these simple bodies however, prevents whatever of inconsistency is attached to their designation from causing much, if any confusion to the student, but when we consider the array of innumerable compounds that are and will be produced, a single and universal term for each, indicating its composition, seems an imperative necessity to an easy recognition of the object we seek: in this particular however, our science has retrograded immeasurably from the simplicity of its early construction.

In 1782, Guytou DeMorveau published the first idea of a chemical nomenclature. Five years later Lavoisier,—to whom is justly assigned the foremost place among the fathers of modern chemistry,—presented to the French Academy an exhaustive report on the subject, setting forth the admirable system which has since borne his name, and which was generally adopted with but few modifications. Berzelius distinguished the binary compounds of the electro-negative elements according as they corresponded to basic or acid oxides: thus with sulphur, he had the basic *sulphuret of potassium*, and the acid sulph*ide* of carbon; and on the same principle, chlor*uret* of potassium, and chlor*ide* of phosphorus, but these distinctions did not serve the purpose intended, inasmuch as they were used indiscriminately and therefore detracted from needed perspicuity.

The different systems of symbolic notation in existence, until the last fifteen years, were an insurmountable obstacle to an agreement on a unity of terms. The system in ordinary use represented a molecule of water as containing one atom each of its constituent elements (H.O.) Its atomic weights corresponded with the so called equivalents; the substitution of one element by another being supposed to take place atom for atom.

The notation known as that of Berzelius was based on the assumption that all elementary gases contained the same number of atoms in equal volumes so that the atomic constitution of a compound corresponds with its constitution by volume, and therefore he represented water as containing two atoms of hydrogen and one atom of oxygen ($H_2O$.)

"Gerharts unitary system" was based like that of Berzelius, on the hypotheses that all simple gases contain equal numbers of atoms in equal volumes. The formula of water in this system is the same as that of Berzelius, but Gerhart more consistently derived his formulae one from the other by substitution, which method some years later, introduced the present received idea of atomic values. The many objections to the unitary system were well founded so long as the term "equivalent" was considered synonymous with atomic weight, and that when one element took the place of another in substitution, the exchange was invariably atom for atom; but when experiments demonstrated the fact that one atom of an element may neutralize two, three, or more units of equivalency, left free by abstraction of a given number of divisible parts, thus satisfying a dyadic, tryadic, or higher résidue, Gerharts remarkable position was fully endorsed. Then following the "milky way" of so much newly discovered truth, came new text books and new editions of the old ones, some with a nomenclature wherein the presence of the electropositive element in a compound must be emphasized by the inevitable *ic*: others partially adopted the new fashion, still adhering fondly to the major portion of the old, while others endeavored to present a system whereby the mere mention of the name of a compound would photograph on your mind the actual relative number of atoms belonging to each of the elements in the molecule, as chromium dioxi-dichloride, ($Cr O_2 Cl_2$). New text books are yearly entering the lists, every writer on the subject appearing independent of any rule, and bringing confusion worse confounded.

Here we have our old friend "Sal Ammoniac," ($N H_4$) alias "muriate of ammonia" transformed into the less euphonious "chlorhydrate of ammonia," "hydrochlorate of Ammonia" "ammoniac chloride" "ammonium chloride," and chloride of ammonium.

How many can recognize that most wonderful compound, "carbonic acid gas" ($CO_2$) when presented to "carbonic anhydride," or even to "carbonic dioxide" or "carbon dioxide?" "Sulphuretted hydrogen" proves that "a rose by any other name would smell as sweet," for it still retains its odor though called "dihydric sulphide," "dihydrogen sulphide" and "sulphydric acid."

And must we relinquish our cherished ideas of the physical character of a salt, by acknowledging the propriety of naming, especially the mineral acids, "hydrogen salts?" and so have for the same body, "nitric acid" ($NO_3H$,) "hydrated nitric oxide" and "hydrogen nitrate" to say nothing of "aqua fortis."

A time honored and convenient method of distinguishing relative quantities of the electronegative present in any two normal compounds made up of the same elements, by ending the name of the lower proportional compound in *ous*, and the higher in *ic* has been carried farther than formerly; thus *ferrous* oxide and ferr*ic* oxide. ($FeO$ and $FeQO_3$) have superceded the more cumbrous "protoxide of iron," and "sesquioxide of iron," the corresponding salts, say of sulphuric acid, being now better known as ferr*ous* sulphate" ($Fe S O_4$,) and *ferric* sulphate ($Fe_3 S O_4$) instead of the respective *proto* and *per*sulphate of iron. But like all good things, this rule has been frequently abused; carbon forms with oxygen two compounds wherein to a given quantity of carbon in each compound is united oxygen in proportions of one and two, ($CO$ and $CO_2$.) Now the monoxide is invariably called "carbonic" rather than according to the rule "carbon*ous*" oxide, and the dioxide generally as carbon*ic acid* gas."

Again in many cases where but one compound is formed by two elements, as in the haloid salts of the monad metals, the terminal *ic* is often misapplied, *vide* potass*ic* iodide (KI,) "sod*ic* chloride (Nacl) etc.; and even silver nitrate puts on the style and becomes argen*tic* nitrate, just as though there were such bodies known as argent*ous* nitrate, Sod*ous* Chlori*de* and potassous iodide. We notice the term "nitrous oxide" is given to the trioxide of nitrogen (N 2 O 3,) probably through a vague idea of propriety because it is the anhydrous binary of nitrous acid (N O 2 H,) notwithstanding the familiar presence of a body two steps lower in the proportional scale, the real true "nitrous oxide" or "dentists laughing gas" (N 2 O.)

It is indeed fortunate that the Pharmaceutical preparations were not often named according to their chemical composition for from the very nature and object of their use it would be extremely dangerous, if not impossible, to twist their designations into compliance with the whims of every self appointed reviser of chemical terms. As it is, our Babel is only fruitful of some inconvenience to the unfamiliar student, and ere' long the muddle that troubles us now, will doubtless be overcome by submission of all rivalries on this subject to a properly constituted Scientific Tribunal, whose fiat will be willingly obeyed, once more enabling white winged Peace to hover o'er the gory field, where 'erst occurred the "Battle of the Books."

